# CMap: a database for mapping job titles, sector specialization, and promotions across 24 sectors

**DOI:** 10.1038/s41597-025-05526-3

**Published:** 2025-07-14

**Authors:** Shehryar Subhani, Shahan Ali Memon, Bedoor AlShebli

**Affiliations:** 1 Computer Science Program, Science Division, New York University Abu Dhabi, Abu Dhabi, United Arab Emirates; 2 Social Science Division, New York University Abu Dhabi, Abu Dhabi, United Arab Emirates; 3 Information School, University of Washington, Seattle, WA USA

**Keywords:** Industry, Databases

## Abstract

Understanding job titles, career trajectories, and promotions provides valuable insight into labor market dynamics and patterns of professional mobility. We introduce Career Map (CMap), a novel, large-scale dataset spanning 24 industry sectors, designed to support the study of job specialization, sectoral concentration, and career advancement. Using natural language processing techniques and large language models, we standardize 5.2 million job titles into 123 thousand unique titles and propose a Specialization Index to quantify how concentrated a given title is within a sector. The dataset includes both a structured job titles dataset and a set of identified promotions—32 thousand validated promotions from the United States and the United Kingdom, and 61 thousand inferred promotions from a global context. CMap enables research on job hierarchies, cross-sector mobility, and systemic inequalities in professional advancement. It provides a foundation for examining how education, experience, and institutional structures shape career outcomes across industries and regions, offering a valuable resource for economists, sociologists, and computational social scientists.

## Introduction

Understanding career trajectories is key to analyzing labor market dynamics, workforce mobility, and the structural organization of job hierarchies. Career trajectories are shaped by a combination of individual qualifications, industry norms, and broader economic conditions, influencing job specialization and promotion pathways across sectors. Tracking how professionals move between roles enables researchers to study occupational mobility, workforce planning, and disparities in advancement opportunities.

Within these career trajectories, job promotions play a crucial role in shaping organizational structures, reflecting individual achievement while redistributing responsibility^1,2^. Understanding how promotions operate across industries and regions is vital for studying career progression, employee development, and labor mobility^3,4^. Researchers and organizations seek to identify promotion pathways to improve workforce planning, talent management, and employee retention, and to address disparities in advancement opportunities^5–8^.

A key factor influencing promotions is job specialization^9,10^, which defines the distinct skills, qualifications, and experiences required for career progression within different industries. Job specialization and promotions are inherently linked, as sector-specific requirements shape career progression and success^10–12^. Specialized roles often have structured career ladders, whereas generalist positions may exhibit more flexible pathways. For example, industries such as information technology exhibit well-defined hierarchies with clear educational and experiential benchmarks, whereas creative sectors often follow less structured career ladders^13^. Understanding how these dynamics operate across industries is essential for evaluating how workers navigate career progression and how organizations structure advancement opportunities.

Despite the importance of job specialization in shaping career mobility, prior research has largely relied on small, sector-specific datasets^13–19^, limiting generalization^20^. Without comprehensive, large-scale analyses, the fragmented nature of these studies has hindered a broader understanding of how specialization influences promotions across different labor markets. Addressing this gap requires datasets that capture career movements across multiple sectors, providing a more holistic perspective on specialization and job trajectories. In recent times, computational advancements have enabled the extraction of job hierarchies from large-scale career trajectory data^21^. Additionally, machine learning techniques have been used to predict career movements based on employment attributes^22^. While such methods might reveal hierarchical structures, they do not draw a distinction between specialized and non-specialized job titles and do not explicitly identify promotions.

To address these challenges, we present Career Map (CMap), a dataset comprised of two parts: (i) job titles and (ii) identified promotions, both globally representative and spanning 24 industry sectors. This dataset is derived from a comprehensive collection of 546 million professional experiences sourced from over 220 million publicly available CVs across 197 countries. The job titles were developed using advanced natural language processing (NLP) techniques and large language models (LLMs) to clean and standardize over 114 million unique job titles. This process reduced the number of titles to 5.2 million, further grouped into 593 thousand standardized titles while preserving contextual relevance. We also introduce a Specialization Index (SI) that reflects how specialized each title is within its sector. Additionally, we provide curated hierarchies of job promotions across various sectors. It includes approximately 32 thousand promotions from the United States and the United Kingdom, as well as approximately 61 thousand promotions from a global context, offering valuable opportunities for exploratory research. These promotions were identified through robust statistical methods and rigorous validation processes, ensuring reliability and consistency. Together, these datasets serve as a comprehensive foundation for analyzing professional trajectories and career advancements across diverse sectors and regions.

The dataset serves two primary functions. First, it offers unprecedented breadth, surpassing prior research constrained by sector-specific or regional limitations. By encompassing diverse job movements, it allows for comprehensive analyses of global labor trends and variations in promotion dynamics across industries. Second, it provides a high-resolution resource for computational researchers developing tools to analyze career progression. With promotions spanning multiple sectors and demographic contexts, it facilitates nuanced analyses of global trends and localized disparities. Rigorous data cleaning and validation ensure comparability across regions and industries, contributing to meaningful insights into career mobility.

Finally, our dataset complements existing resources such as the Occupational Information Network (O*NET)^23^, which catalogs occupations, job titles, tasks, required skills, and other labor market insights. While O*NET provides structured occupational taxonomies at the Standard Occupational Classification (SOC) level, our dataset retains title-level specificity, preserving distinctions in seniority, sectoral alignment, and specialization. It includes crosswalks to SOC codes and is designed for integration with external datasets such as wage records (e.g., Glassdoor, national statistics), demographic surveys (e.g., ACS, IPUMS), and job postings (e.g., LinkedIn, Indeed), making it well-suited for interdisciplinary labor market research. Researchers can tailor analyses by adjusting promotion thresholds, selecting from multiple levels of title granularity, or isolating specialization dimensions such as sector exclusivity and dominance. In addition to descriptive and comparative analyses, the dataset supports predictive modeling—enabling researchers to estimate promotion likelihoods, simulate career pathways, and identify structural barriers to advancement. This modular architecture empowers the development of scalable, data-driven insights into labor mobility, job hierarchies, and systemic inequality across global labor markets.

## Methods

We generated our dataset by utilizing a collection of 220 million anonymized and publicly available user curriculum vitae (CVs), collected from LinkedIn via DataHut^24^. These CVs encompass a total of 546 million job experiences spanning 197 countries and 24 industry sectors. While the dataset itself was collected in 2017, the career histories recorded within these CVs extend as far back as 1970, capturing job trajectories of individuals whose professional experiences span multiple decades, up to December 2017. However, given the noisy nature of the data, substantial pre-processing was required to ensure consistency and usability. The job titles appeared in a variety of formats, often containing spelling variations, abbreviations, or redundant information. Furthermore, sector classifications were inferred rather than explicitly provided, requiring additional processing to associate each job experience with a standardized industry category. The methods outlined in this section describe the steps taken to clean and structure the dataset to facilitate meaningful analysis of job transitions and promotions across different sectors and geographic regions.

### Data Cleaning and Filtering

**Fig. 1:**
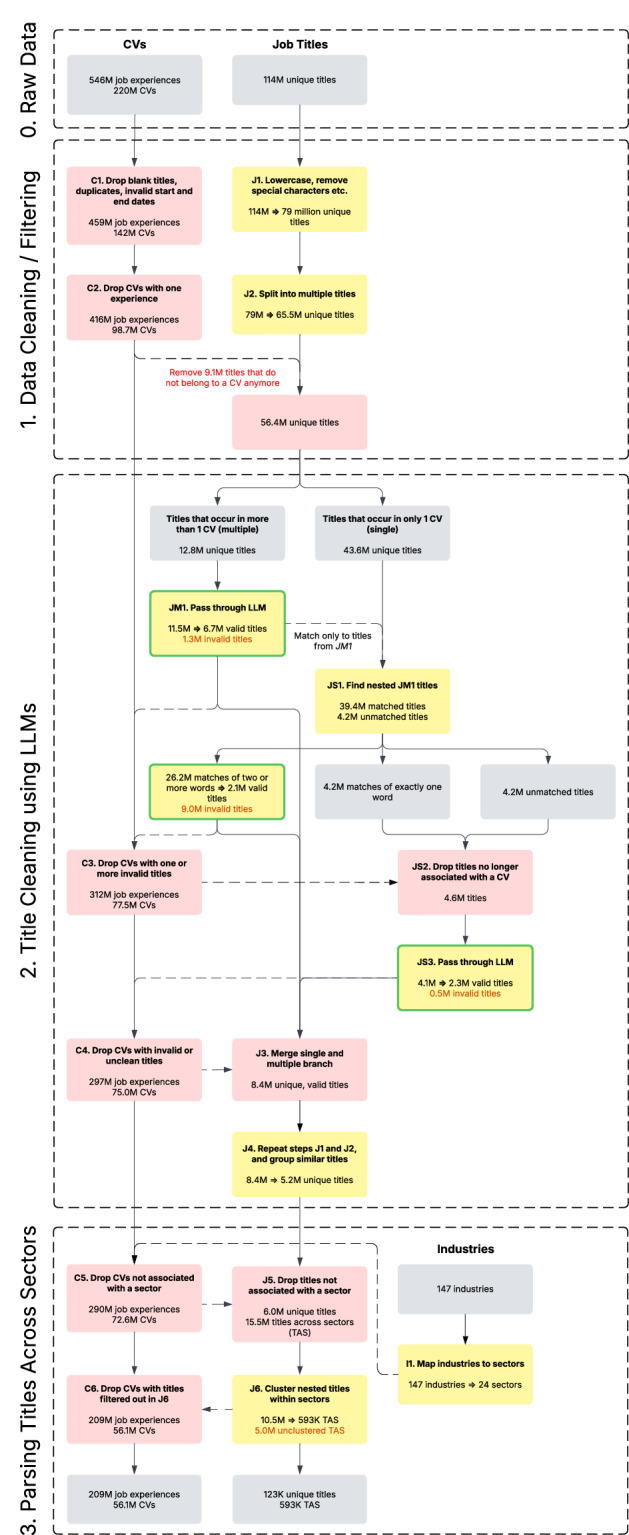
The data cleaning process of job titles. Grey boxes represent input or intermediate data descriptions. Red boxes represent data removal or filtering. Yellow boxes represent data modification. Green borders represent the use of LLMs.

The data cleaning process began with the simultaneous cleaning of CVs and job titles, as illustrated in Fig. 1. Job titles underwent a thorough cleaning and filtering process (labeled as J1 in Fig. 1), which included removing content within brackets, non-alphanumeric characters, single-letter words, and unnecessary spaces or special characters at the boundaries of titles. These normalization steps standardized job titles, reducing the number of distinct titles from 114 million to 79 million while preserving the total number of job experiences. Additionally, we addressed cases where users listed multiple job titles within a single experience, such as “freelance translator and interpreter” (labeled as J2 in Fig. 1). To handle such cases, we split composite titles using common separators like commas and applied part-of-speech tagging to identify nouns and conjunctions. For example, “freelance translator and interpreter” was split into “freelance translator” and “freelance interpreter.” This step further reduced the number of distinct job titles to 65.5 million.

For CVs, we removed job experiences that lacked essential information such as job title, start date, end date, or organization. Duplicate job experiences—those with identical start dates, end dates, organizations, and job titles—were also eliminated to enhance data accuracy (labeled as C1 in Fig. 1). Additionally, to analyze job hierarchies, we focused on CVs containing multiple job experiences and excluded CVs with only a single experience (labeled as C2 in Fig. 1). This filtering step was essential to ensure the validity of promotion identification and trajectory-based metrics. As a result, the dataset was reduced to approximately 98.7 million CVs. In the process, 9.1 million unique job titles (previously associated only with the excluded single-experience CVs) were also removed. This brought the total number of distinct titles down to 56.4 million.

### Title Cleaning using Large Language Models (LLMs)

Large language models (LLMs) like GPT-3.5 and GPT-4 excel at a wide range of natural language tasks, including question answering^25^, entity recognition^26^, relation extraction^27^, summarization^28^, machine translation^29^, paraphrasing^30^, and even various data wrangling tasks^31^. Their ability to perform *in-context learning* enabled them to adapt to new tasks without retraining or updating their parameters^32^. Given properly structured prompts—and sometimes additional fine-tuning or few-shot examples—LLMs can identify whether a string looks like a legitimate job title and then reformat or simplify it. Their extensive pretraining on vast, diverse datasets and large parameter sets equips them to recognize semantic equivalence across various job titles. For instance, they can equate “Asst Mgr” with “Assistant Manager” and remove extraneous details such as geographic indicators or employer names, thereby mapping various forms of the same role to a single, concise version.

We began by dividing the dataset of 56.4 million unique job titles into two groups: those that appeared in multiple CVs and those that appeared in only one CV. This division was based on the assumption that the more frequently a title appears across job experiences, the more likely it is to be a valid job title. At this stage of the data cleaning process, 43.6 million out of the 56.4 million titles occurred only once in the entire dataset. These infrequent titles included over-descriptive entries such as “assistant manager at Microsoft in India” and invalid titles like “swimming pool.” In contrast, common job titles such as “owner” and “project manager” appeared 4.5 million and 3.4 million times, respectively.

**Fig. 2:**
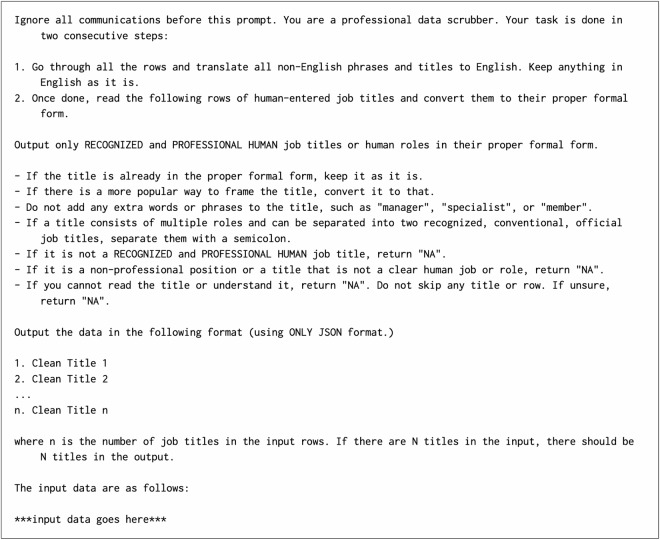
The prompt used by LLM for processing titles in steps *JM1* and *JS3* as shown in Fig. 1. The titles are shuffled and assigned to a random group of up to 50 titles before being passed onto the LLM. Each prompt is independent of the preceding prompts.

To refine the more frequent titles, we employed *gpt-4-turbo-preview* to evaluate and standardize them. Titles that appeared in two or more job experiences were passed on to the LLM, which had two primary objectives: (i) determine whether a given title was a valid job title and (ii) if valid, to standardize the title into its most concise, non-abbreviated, professional form. For example, when presented with the title “swimming pool,” the LLM returned “NA,” indicating it was invalid. Conversely, for a title like “assistant manager at Microsoft in India,” the LLM simplified it to “Assistant Manager.” The specific prompt used for this task is shown in Fig. 2. Of the 12.8 million titles processed by the LLM, 1.3 million were identified as invalid, while the remaining 11.5 million underwent simplification. This step reduced the total number of unique job titles from 11.5 million to 6.0 million, further streamlining the dataset (labeled as JM1 in Fig. 1).

Given the substantial number of unique titles that appeared only once (43.6 million), we opted to filter these singleton titles before subjecting them to LLM evaluation (labeled as JS1 in Fig. 1). These titles were compared with the titles already processed by the LLM in the preceding step (JM1). The goal was to identify whether a singleton title contained a subset that matched a clean title from JM1. If such a match was found, we mapped the singleton title to the LLM-generated output of the matching title. For instance, the title “accountant state bank” is a literal subset of “accountant state bank pvt ltd,” allowing us to map the latter to the LLM-generated title “State Bank Accountant.” Our algorithm prioritized matches based on the length of the matching title, with longer titles given precedence. In rare instances where multiple matches of the same length were found, the algorithm considered the frequency of those titles in the dataset, prioritizing the most frequent title. Out of the 43.6 million singleton titles, 39.4 million were successfully matched: 26.2 million were mapped to clean titles containing two or more words, 4.2 million to clean titles consisting of a single word, and 9 million to titles identified as invalid in JM1. The 26.2 million titles mapped to multi-word LLM-generated titles were further condensed into 2.1 million unique titles from the previous step. Meanwhile, the 4.2 million titles mapped to single-word LLM-generated titles were flagged for reassessment, as these mappings might have been overly generalized. For example, the title “excutve assistant” would be correctly identified as “Executive Assistant” instead of “Assistant,” ensuring greater accuracy.

At this stage, we were left with 4.2 million unmatched titles and 4.2 million titles mapped to overly generalized one-word titles. To further minimize this number and improve data quality, we eliminated all CVs containing one or more invalid titles identified in steps JM1 and JS1 (labeled as C3 in Fig. 1). This decision was motivated by two key concerns: (i) removing invalid titles often left users with only a single valid job experience, precluding the construction of job hierarchies, and (ii) if an invalid title appeared in the middle of a CV, retaining the surrounding job titles would lead to inaccurate inferences about career progression. As a result, this step reduced the number of CVs from 98.7 million to 77.5 million. Consequently, any job titles no longer associated with a job experience were discarded, reducing the remaining 8.4 million job titles to 4.6 million. These remaining titles were then re-evaluated using the LLM from step JM1 (labeled as JS2 in Fig. 1). During this round of evaluation, 0.5 million titles were flagged as invalid, while the remaining 4.1 million titles were simplified, resulting in a further reduction to 2.3 million unique titles. By combining the clean and valid titles from steps JS1, JS3, and JM1, we finalized a dataset of 8.4 million unique job titles (labeled as J3 in Fig. 1). These titles were derived from a dataset comprising 75 million CVs, covering a total of 297 million job experiences (labeled as C4 in Fig. 1).

Next, we repeated Step J2 on the latest set of job titles, as some previously uncaptured hybrid titles could now be separated into distinct titles following the LLM cleaning. Additionally, since certain abbreviations might have multiple potential expansions or exist only in their abbreviated form within our dataset, we chose not to expand them during the initial LLM cleaning phase. Instead, in this step, we addressed abbreviation expansion by searching for their full forms within the dataset. For instance, for the title “IT Manager,” we identified job titles where the first word started with “I,” the second with “T,” followed by “Manager.” If multiple matches were found, the most frequent variant was selected. Furthermore, we standardized titles containing prepositions, such as converting “Director of Finance” to “Finance Director.” These additional steps improved the consistency of the dataset, reduced variations of the same title, and further streamlined the dataset, reducing the total number of unique titles to 5.2 million (J4 in Fig. 1).

Finally, before proceeding with parsing titles into different sectors (as discussed in the next section), we validated the effectiveness of our title-cleaning methodology using the LLM across all stages where its outputs were directly used or merged with other LLM-processed datasets—namely stages JM1, JS1, JS3, and J3. These validations focused on two core tasks: (i) identifying whether a string constituted a valid job title, and (ii) standardizing it into a concise, professional form. Our evaluation demonstrated high accuracy, precision, and recall across these steps (see “Title Cleaning Validation” under the “Technical Validation” section). Importantly, multilingual samples (primarily in Spanish and French) were proportionally included in this validation, and we observed no significant decline in performance relative to English titles. While the final dataset is fully standardized into English, approximately 7% of job titles were originally submitted in a non-English language, closely mirroring the 9% share in the raw data. This suggests that the title-cleaning pipeline performs reliably across languages and does not introduce systematic exclusion or loss of non-English entries.

### Parsing Titles across Sectors

Each CV was initially assigned to one of 147 predefined industries, which were subsequently mapped to 24 broader sectors. The sector of each CV was determined based on its most recent job experience, with CVs lacking a predefined industry excluded. This refinement reduced the dataset to 72.6 million CVs and 6.0 million unique job titles (see C5 and J5 in Fig. 1). Moving forward, titles will be treated separately within each sector. This is to address variations while accounting for titles like “Receptionist” or “Intern” that may appear in multiple sectors. The aim is to parse titles within each sector by identifying which titles belong to which sector accordingly. Furthermore, to better track data retention, we will report both the number of unique titles retained after each step and the total number of titles across all sectors (TAS). For example, if “Receptionist” appears in 15 sectors, it will count 15 times in TAS but only once in the unique titles count.

Until now, job titles have been preserved as submitted by users as much as possible, resulting in 6.0 million distinct titles (15.5 million titles across sectors), which is an unreasonably large number for establishing a hierarchy within each sector. To streamline this, we generalize titles within each sector by dividing them into two groups: (i) common titles, defined as the top 5% most frequent titles in the sector that also appear more than 100 times in the entire dataset, and (ii) all other titles. Using the first group as a reference, we identify subset matches in the second group within each sector (similar to step JS1), prioritizing longer titles and selecting the highest-frequency match when multiple options exist. For example, “Technical Electromechanics Professor” is clustered into “Technical Professor”. To avoid overgeneralization, we (i) exclude one-word titles like “Manager” from clustering and (ii) preserve keywords that indicate seniority or responsibility to maintain job hierarchies.

Of the 15.5 million titles across sectors, 10.5 million were successfully clustered within their sector by matching titles in the first group. This reduced the total number of titles across sectors to approximately 593 thousand (see J6 in Fig. 1). However, 5.0 million titles remained unclustered due to the difficulty of aligning these rare titles with more common ones within the sector. As a result, these titles, along with their corresponding CVs, were excluded. This exclusion led to reduction of 16.5 million CVs (see C6 in Fig. 1).

**Fig. 3:**
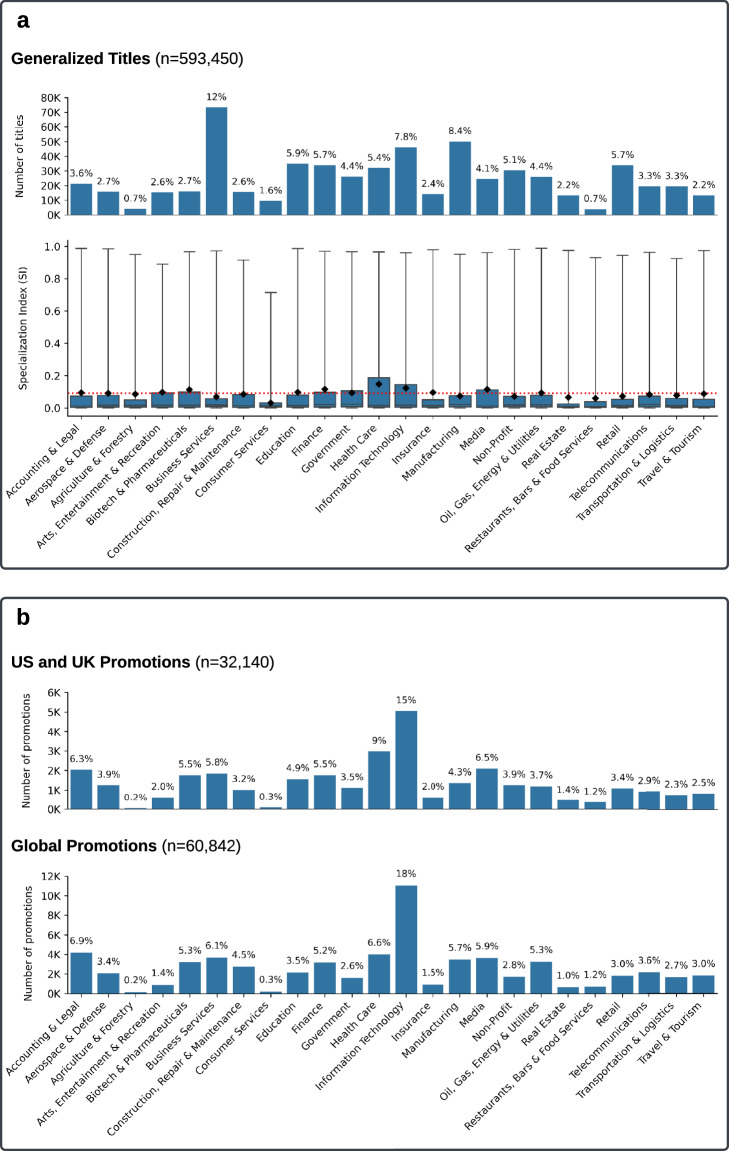
Overview of the dataset. (**a**) The top plot shows the distribution of generalized job titles, while the bottom plot presents the Specialization Index (*SI*) distribution by sector. Whiskers indicate the range of *SI* scores, with the black diamond marking the sector mean. The dotted red line represents the overall mean threshold for specialized titles (*SI* = 0.11). (**b**) The distribution of promotions by sector and region.

The final dataset comprises approximately 593 thousand job titles across 24 sectors, derived from over 123 thousand unique titles. For an overview of the dataset distribution across sectors, please refer to the top plot in Fig. 3a. To facilitate further research, we provide this dataset as the first part of a resource for other researchers (see Data Records). Additionally, we include the “cleaned” version of each title prior to generalization (J5), and the “generalized” version (J6). The resulting dataset contains 10.5 million job titles mapped to their corresponding 593 thousand generalized titles across 24 sectors. Table 1 provides examples from each of the top 10 sectors, illustrating the data before and after the cleaning process.

**Table 1:** Examples of Original and Generalized Job Titles Across the Top 10 Sectors.

Sector	Original Title	Generalized Title(s)
Business Services	Sr. Mktng Specilist / Sales rep	[Marketing Specialist, Sales Representative]
Manufacturing	Prod. Engr.	Production Engineer
Information Technology	Sftwre Eng / Développeur microsoft	[Software Engineer, Software Developer]
Education	Adjunct Prof.	Adjunct Professor
Retail	Store asst.	Store Assistant
Finance	Boekhouder	Bookkeeper
Health Care	Regstrd Nurse	Registered Nurse
Non-Profit	fundraising mngr.	Fundraising Manager
Government	civil servnt	Civil Servant
Oil, Gas, Energy & Utilities	Petroleum Eng. / Refinery Oprtr.	[Petroleum Engineer, Refinery Operator]

This table illustrates the outcome of the cleaning process, where original job titles have been standardized into generalized titles to ensure consistency and comparability across sectors.

Finally, to balance precision with flexibility, we chose to preserve nuanced distinctions between job titles and maintain as much of the original title integrity as possible. For instance, while titles such as ‘Postdoctoral Fellow,’ ‘Postdoctoral Associate,’ and ‘Postdoctoral Research Fellow’ may appear interchangeable—particularly in academia, where naming conventions vary across institutions and countries—we retained them as distinct entries. This decision reflects our commitment to avoiding assumptions about title equivalence in the absence of consistent standards or sector-specific domain expertise. Similarly, although some may consider titles like ‘Sociology Assistant Professor’ and ‘Economics Assistant Professor’ to represent equivalent roles, we preserved the disciplinary distinctions to give users the flexibility to tailor aggregations based on their specific research objectives. For example, a researcher studying early-career academics might choose to group all postdoctoral roles using keyword-based filters.

To support such use cases, we include a ‘simplified’ version of each title in the dataset, offering an optional reference layer for those seeking more standardized groupings. However, we caution that this layer may occasionally overgeneralize—for example, collapsing ‘Accounting Manager’ and ‘Finance Manager’ into a generic ‘Manager.’ We leave these aggregation decisions to the discretion of the user. Moving forward, and in our own analyses, we rely on the ‘generalized’ version of job titles, which retains structural and sectoral information but can easily be linked to the simplified titles if users wish to conduct higher-level analyses.

### Creating a Specialization Index

A notable limitation, however, in associating sectors with CVs is that the assigned sector typically reflects only the user’s most recent experience. As a result, if a user has transitioned between sectors over their career, all job titles on their CV will be linked to the sector of their most recent role. While earlier steps in J6 help mitigate this issue, some job titles may still appear in sectors where they do not intuitively fit. For instance, a title like “receptionist” could be relevant across various industries.

To address this, we introduce a metric, called the *Specialization Index* (*SI*), to quantify the degree to which a job title is specialized within a particular sector. The aim of this metric is to evaluate how concentrated a title is both across sectors and within a specific sector, allowing us to distinguish between roles that are more “specialized” versus “generalized”. The *SI* combines two complementary components, each capturing a distinct dimension of specialization:

1. *Sector Exclusivity* (*SE*): based on the Herfindahl-Hirschman Index^33^, this measure captures how narrowly distributed a title is across sectors. A score of 1 indicates that the title is exclusively associated with a single sector, while a score closer to 0 suggests that the title is broadly distributed across multiple sectors. More formally, let *f*_*t*,*s*_ denote the frequency of title *t* in sector *s*, and let *T* and *S* represent the sets of all titles and all sectors, respectively. Because sectors vary in overall size, we compute a weighted frequency for each title-sector pair as:
  $${f}_{t,s}^{* }=\frac{{f}_{t,s}}{{w}_{s}}$$
  where *w*_*s*_ is the weight of sector *s*:
  $${w}_{s}=\frac{{\sum }_{t\in {T}_{s}}{f}_{t,s}}{{\sum }_{t\in T}{\sum }_{s\in S}{f}_{t,s}}$$
  and *T*_*s*_ is the set of all titles in sector *s*. If a title *t* is not present in sector *s* (i.e., if *t* ∉ *T*_*s*_), then *f*_*t*,*s*_ = 0. The Sector Exclusivity for title *t* is then given by:
  $$S{E}_{t}=\sum _{s\in S}{(\frac{{f}_{t,s}^{* }}{{\sum }_{s\in S}{f}_{t,s}^{* }})}^{2}$$
  This measure captures the degree of cross-sector specialization, with higher values indicating greater exclusivity to a particular sector. For example, ‘Instructional Coach’, a job title predominantly found in the ‘Education’ sector, has a high *SE* of 0.80. On the other hand, a job title such as ‘Manager’ which is found in all sectors has a low *SE* of 0.05.
2. *Sector Dominance* (*SD*): This metric captures how prevalent a job title is within a specific sector. It combines two sub-components that reflect both intra-sector and cross-sector prominence. First, we calculate the *Frequency Percentile* (*FP*) of title *t* in sector *s*:
  $$F{P}_{t,s}=\frac{rank({f}_{t,s}^{* })}{| {T}_{s}| }$$
  where $rank({f}_{t,s}^{* })$ is the rank (in ascending order) of *t*’s weighted frequency among all titles in sector *s*, and ∣*T*_*s*_∣ is the number of unique titles in that sector. This percentile indicates how frequent the title is relative to its peers, offering insight into its internal prominence or commonality. As such, infrequent titles within a sector will have a low *FP* (closer to 0) while frequent titles will have a higher *FP* (closer to 1). For example, in the ‘Healthcare’ sector, a relatively uncommon title such as ‘Registered Nursing Student’ has an FP of 0.68 (or 68*th* percentile) while ‘Physiotherapist` has an *FP* of 0.999 (or 99.9*th* percentile).

Next, we compute the *Ratio to Maximum Frequency* (*RMF*):

$$RM{F}_{t,s}=\frac{{f}_{t,s}^{* }}{{f}_{t,{s}_{\max }}^{* }}$$

where ${s}_{\max }$ is the sector where title *t* has its highest weighted frequency across all sectors. This ratio provides a cross-sectoral perspective on how dominant the title is in sector *s* relative to its strongest association elsewhere. In this case, a title *t* with an *RMF*_*t*,*s*_ of 1 would have the most occurrences of *t* in its sector *s*. For example, ‘Professor’ has an RMF of 1 in ‘Education’ (where it occurs the most) but an RMF of 0.11 in ‘Business Services’. This suggests that the title ‘Professor’ occurs approximately 10 times more in ‘Education’ than it does in ‘Business Services’.

The Sector Dominance is then defined as the product of these two terms:

$$S{D}_{t,s}=F{P}_{t,s}\times RM{F}_{t,s}$$

A value of 1 indicates that the title is both highly common in the sector and represents its peak frequency across all sectors. In contrast, a score closer to 0 indicates minimal presence.

Combining these two dimensions, we define the Specialization Index (*SI*) as:

$$S{I}_{t,s}=S{E}_{t}\times S{D}_{t,s}$$

A title attains a high *SI* (closer to 1) when it is both narrowly distributed across sectors (high *SE*) and deeply embedded within one sector (high *SD*). While *SI* captures the combined notion of specialization, we also provide its two underlying components, *SE* and *SD*, independently with each job title, to give researchers flexibility in examining either dimension on its own. *SE* is more appropriate when comparing how broadly a title is distributed across sectors, whereas *SD* is better suited for analyzing its prominence within a specific sector. It is important to note, however, that since SI is a composite of SE and SD, an SI of 0.30 is not necessarily “three times” more specialized than 0.10, and should not be interpreted in percentage terms, as they reflect relative, and not absolute, specialization.

Furthermore, this comprehensive formula allows us to categorize job titles within a sector as either ‘generic’ or ‘specialized’. Moving forward, we opted to use the mean *SI* as the threshold to distinguish between specialized and generic titles (see J7 in Fig. 1). As such, if a title has an *SI* greater than the global mean in one sector, it is classified as “specialized” within that sector, and all instances of that title in other sectors are reassigned to this sector. If all occurrences of the *SI* of a title across all sectors are less than the mean, it is classified as “generic.” For example, in the “Information Technology” sector, “Manager” has an SI of 0.01 (generic) while “Senior Salesforce Developer” has an SI of 0.91 (specialized). For an overview of the SI distribution within each sector, please refer to the bottom plot in Fig. 3a, where the red line indicates the mean threshold used.

### Forming Job Hierarchies

**Fig. 4:**
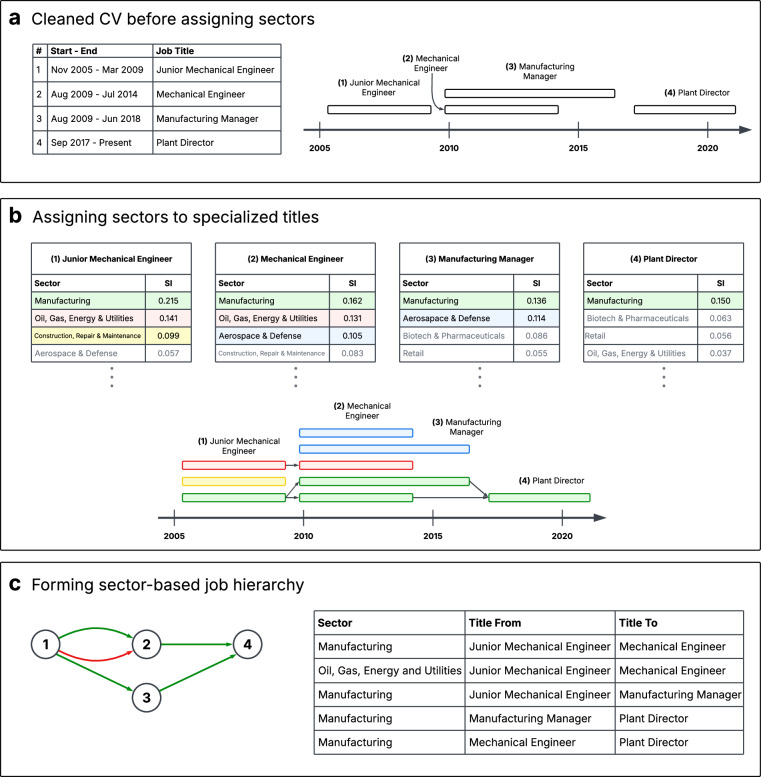
Overview of job trajectory mapping and sector assignment. (**a**) A cleaned CV dataset displaying job titles and corresponding employment periods, along with a timeline visualization of the career progression. (**b**) Job titles are categorized into relevant sectors based on their Specialization Index (SI), accompanied by a visual representation of sector assignments over time. This visualization highlights transitions within each sector, with distinct colors assigned to differentiate between sectors. (**c**) A schematic diagram and table illustrating job movements within each sector.

A career trajectory maps an individual’s professional growth over time, encompassing their sequence of roles from entry-level positions to senior leadership, as well as transitions across industries or functions. To ensure our analysis captures meaningful within-sector career advancements, we focus exclusively on highly specialized job transitions within each sector. Using the Specialization Index we introduced earlier, we include only transitions above the mean with a score of 0.092 or above. This threshold ensures that the analysis highlights transitions reflecting the highest degree of sector-specific expertise, which are more likely to signify significant career milestones. To identify promotions within specialized career transitions, we construct job hierarchies at the individual level by mapping directional edges between consecutive job experiences within each CV. These edges represent career transitions and are assigned based on the chronological order of job positions. First, job titles and their corresponding employment periods are structured to ensure consistency in analyzing career trajectories. This step organizes career histories into a format that allows for systematic examination of job movements over time (see Fig. 4a). Next, each job title is assigned to relevant industry sectors based on their Specialization Index (SI). Finally, directional edges are established between non-overlapping job transitions to form a sector-based job hierarchy (see Fig. 4b). These transitions link lower-level job titles to higher-level roles, allowing us to model career advancement within and across sectors. The resulting hierarchy provides a structured representation of job mobility, capturing both sector-specific career ladders and broader inter-industry movements (see Fig. 4c).

The individual-level data is then aggregated at the continent-sector level, resulting in job transitions across 144 continent-sector combinations. For each transition, several key characteristics are tracked: the average years of education individuals had before starting the job, the average years since the start of their career, and, for select countries, the average salary. Additionally, the total number of individuals who made that job transition during their career is recorded. These characteristics are used to calculate four metrics that help determine the likelihood of a promotion, providing a comprehensive framework for assessing whether a transition represents a promotion or not.

The first metric focuses on the role of education in career progression, which is widely recognized as a key factor in signaling an individual’s potential capability^34,35^ and readiness for more senior roles^36^. Studies also show that organizations frequently use education as a screening tool in promotion decisions, further underscoring its importance^36^. To capture this, we introduce the Education Progression Score (EPS), which quantifies the extent to which a job transition reflects a statistically significant increase in educational attainment. To calculate EPS for a job transition from job A to job B, we use the International Standard Classification of Education (ISCED)^37^ to rank the highest degrees held by individuals who have made this transition, converting these ranks into equivalent years of education (e.g., 12 years for a high school diploma, 18 years for a master’s degree). For each job title, we compute the mean and standard deviation of years of education among its holders within the same continent-sector combination. Welch’s t-test is then used to assess whether the mean years of education significantly increase from job A to job B for individuals making this transition. The EPS is then defined as 1 − *p*, where *p* is the p-value from the t-test. This provides a continuous measure of educational progress, with EPS values ranging from 0 to 1. Higher values (closer to 1) indicate a significant increase in the average education required for the transition from job A to job B, while lower values (closer to 0) suggest no significant increase in required education.

Similarly, the Job Start Score (JSS) evaluates whether a job transition corresponds to a statistically significant progression in the typical time taken to reach a position within a career. Prior research indicates that career progression often depends on the accumulation of experience and skills over time^38,39^, with years of work experience acting as a proxy for readiness to assume more complex responsibilities^40^. Therefore, we theorize that relatively senior roles require more years of work experience and hence occur later in a person’s career. To evaluate this, we calculate the mean and standard deviation of the time elapsed from the start of an individual’s career (as documented in their CV) to when they assume a given job title, within each continent-sector combination. A Welch’s t-test is then applied to determine whether there is a significant increase in the mean time required from one job to the next within a specific job transition. The JSS is defined as 1 − *p*, where *p* is the p-value from the Welch’s t-test. This provides a continuous measure of career progression, with values ranging from 0 to 1. Higher JSS values (closer to 1) indicate a significant increase in the years of experience required to transition from job A to job B, whereas lower values (closer to 0) suggest no meaningful difference in the required experience.

Next, we calculate the Edge Proportional Difference Ratio (EPDR), which measures the directional imbalance of transitions between two positions in a job transition. We theorize that if significantly more individuals move from job A to job B than from job B to job A, the dominant direction is more likely to represent a promotion. The EPDR is therefore defined as:

$$EPDR=\frac{{f}_{A\to B}\,-\,{f}_{B\to A}}{{f}_{A\to B}+{f}_{B\to A}}$$

where *f*_*A*→*B*_ represents the number of individuals who transitioned from job A to job B at some point in their career, and *f*_*B*→*A*_ represents the number of individuals who transitioned from job B to job A. Values closer to 1 indicate that the majority of individuals are moving from job A to job B, while values closer to  − 1 suggest the opposite.

Similarly, the Salary Proportional Difference Ratio (SPDR) compares the average salaries between two positions in a job transition, adjusted for their respective countries. Salary is widely recognized as a key determinant of promotions, with studies emphasizing that promotions typically result in substantial wage increases^34^, reflecting the added responsibilities^41^ and skills required for senior roles^1,2^. Consequently, we hypothesize that relatively senior roles are associated with higher salaries. The SPDR is therefore calculated as:

$$SPDR=\frac{{S}_{A}\,-\,{S}_{B}}{{S}_{A}+{S}_{B}}$$

where *S*_*A*_ is the median salary job A in the respective country and *S*_*B*_ is the median salary job B in the respective country. SPDR is calculated only for the United States and the United Kingdom, as these two countries have the highest number of job transitions in the dataset. The salary data was sourced from RapidAPI’s Job Salary Data API^42^, which compiles average salary estimates for various job titles using publicly available job postings from websites such as Glassdoor and Indeed. The API also provides salary benchmarks of a specific country in its local currency, facilitating a more accurate comparison of salary differentials between job transitions within its respective country.

Finally, we estimate the probability of a job transition being a promotion using a logistic regression model that incorporates the four key variables, along with sector and country fixed effects. The model is trained on job pairs ranked in the top 10% of each sector (in terms of frequency), identified as promotions based on the semantic structure of their titles. For example, a transition from ‘Junior […]’ to ‘Senior […]’ is categorized as a promotion, while the reverse is considered a demotion. Using this approach, we identify 1,997 promotions and 625 demotions in the United States, and 804 promotions and 199 demotions in the United Kingdom. The logistic regression results are presented in Table 2. Model (5) is used in predicting whether a job transition constitutes a promotion in the United States or the United Kingdom, demonstrating high reliability. Under a 5-fold cross-validation framework, the model achieves a balanced accuracy of 97.4%, precision of 98.7%, and recall of 99.1% under a 5-fold cross-validation framework. Additionally, the model attained a micro F1 score of 98.3%, highlighting its strong generalization across the dataset. This model is applied to predict the promotion probability of over 48 thousand job transitions in the United States and United Kingdom. Transitions with a probability of 0.9 or higher are classified as promotions, resulting in a final set of 32 thousand promotions across 24 sectors (see Data Records). These classifications are further validated through manual annotations (for details, see Promotions Validation).

**Table 2:** Logistic Regression Results for Promotion Identification.

	*Log-Odds of Promotion*					
	(1)	(2)	(3)	(4)	(5)	(6)
Education Progression Score	1.225^***^				−0.555^**^	−0.365^**^
	(0.047)				(0.179)	(0.119)
Job Start Score		2.951^***^			1.334^***^	0.955^***^
		(0.096)			(0.168)	(0.116)
Edge Proportional Difference Ratio			5.608^***^		4.764^***^	4.553^***^
			(0.305)		(0.343)	(0.234)
Salary Proportional Difference Ratio				−0.281^***^	0.066	
				(0.046)	(0.154)	
Constant	1.491^***^	2.810^***^	4.081^***^	1.180^***^	4.080^***^	4.112^***^
	(0.059)	(0.141)	(0.241)	(0.047)	(0.265)	(0.202)
Sectors	*✓*	*✓*	*✓*	*✓*	*✓*	*✓*
Countries (ref=United States)	*✓*	*✓*	*✓*	*✓*	*✓*	
Continent (ref=North America)						*✓*
Observations	3,625	3,625	3,625	3,625	3,625	7,538
Pseudo *R*^2^	0.219	0.755	0.894	0.013	0.913	0.912

The table presents the estimated log-odds of a job transition being classified as a promotion based on various predictors. Models (1)–(5) report different subsets of the key variables for the validated dataset (US and UK), while Model (6) reports key variables for the unvalidated dataset (the rest of the world). Standard errors are reported in parentheses.

Note:^*^p < 0.05; ^**^p < 0.01; ^***^p < 0.001

This process is also repeated across all continents, excluding salary as a dependent variable due to the lack of accurate salary data outside the United States and United Kingdom. This approach provides an additional 61 thousand job promotions across 24 sectors and 6 continents, providing broader applicability while maintaining accuracy (see Data Records). For the regression results, see Model (6) in Table 2, which also achieved a high balanced accuracy of 97.5%, precision of 98.9%, and recall of 99.2% under a 5-fold cross-validation framework. Additionally, the model attained a micro F1 score of 98.5%, further demonstrating its robustness.

**Fig. 5:**
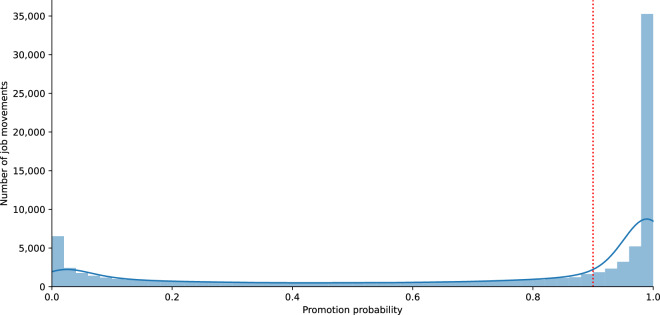
Distribution of job transitions by predicted promotion probability. The histogram shows the frequency of job transitions at different probability levels, while the blue line represents the kernel density estimate (KDE), highlighting underlying patterns. The red dashed line at *x* = 0.9 indicates the threshold used to classify a job transition as a promotion.

In this study, we classify job transitions as promotions if their predicted probability is 0.9 or higher. This threshold is selected based on the distribution of predicted probabilities, which exhibits a bimodal shape, with most values concentrated near 0 or 1 (see Fig. 5). Given this distribution, users of the dataset have the flexibility to choose an alternative probability threshold based on their specific needs. A higher threshold increases the likelihood that the classified transitions represent genuine promotions. Notably, the probability distribution of the alternative model follows a similar pattern. For an overview of the distribution of the promotions datasets across sectors and countries/continents, kindly refer to Fig. 3b.

## Data Records

The dataset used in this study is publicly available on Zenodo^43^ at [10.5281/zenodo.15260189]. It is structured into clearly labeled directories reflecting the key components of the data, job titles and promotions, with further segmentation by sector and region to ensure clarity, modularity, and ease of use:

1. **Job Titles**
  dataset/titles/map: Contains 24 CSV files, one for each sector, listing 593 thousand cleaned, generalized, and simplified job titles with frequency counts. These mappings help standardize noisy job titles across datasets.
  dataset/titles/si: Includes details on weighted frequency, Sector Exclusivity (SE), Sector Dominance (SD), and Specialization Index (SI) for each job title in each sector. Each file also links to O*NET SOC codes, enabling linkage to occupational taxonomies maintained by the U.S. Department of Labor.
  dataset_ext/map-raw: Offers a step-by-step mapping from the original 56.4 million raw titles (post-step J2) to the 109,000 standardized titles (step J7 in Fig. 1). This extended mapping supports broader integration with external datasets (e.g., job postings) and can be used as training data for supervised machine learning models such as SVMs or transformer-based cleaners.
2. **Promotions**
  dataset/promotions/unvalidated: Contains 48 CSV files—24 sectors for each of the United States and the United Kingdom. These files document approximately 32 thousand validated job transitions, manually labeled as promotions by domain expert annotators.
  dataset/promotions/unvalidated: Includes 144 CSV files—24 per continent across Africa, Asia, Europe, North America, Oceania, and South America—documenting approximately 61 thousand statistically inferred promotions.
    Each file also includes computed metrics such as Education Progression Score (EPS), Job Start Score (JSS), and promotion probability.
    As a complementary component to the promotions data, the dataset includes interactive job promotion network visualizations housed in:
    dataset/promotions/validated/network/
    dataset/promotions/unvalidated/network/

These HTML-based visualizations allow users to explore sector and region specific promotion networks. They allow users to explore job flows, key nodes, common transitions, and structural bottlenecks in career progression.

### Data Format and Examples

All files follow a consistent naming format: <REGION>_<sector>.csv, which facilitates straightforward filtering and analysis by country/region or sector.

Finally, an additional examples/ directory provides examples of integrating CMap with:

- American Community Survey (ACS) Public Use Microdata Sample (PUMS) data^44^
- LinkedIn job postings^45^

For a better understanding of the distribution of our data, please refer to Fig. 3a for distributions of title specialization and Fig. 3b for promotion distributions by sector and region.

## Technical Validation

In this section, we outline the validation techniques employed to assess and ensure the quality of both parts of our dataset: (i) the job titles, and (ii) the identified promotions.

### Title Cleaning Validation

We validate the quality of the data produced after each LLM-related cleaning step, JM1, JS1, JS3, and J3 in Fig. 1, across two core tasks:

1. Job Title Identification: determining whether a given job title should be considered valid or invalid.
2. Job Title Standardization: for valid titles, assessing whether the cleaned version preserves the core meaning, hierarchical level, and specificity of the original.

To evaluate the effectiveness of our title cleaning pipeline, we designed a validation procedure involving human annotators. At each of the four stages, we randomly sampled 1,000 job title pairs, where each pair consisted of a pre- and post-cleaning version of the same title. Because each cleaning stage generated a distinct sub-dataset, separate validation was necessary to ensure that transformations at each step maintained high quality.

Each title pair was independently assessed by three trained annotators, who marked each transformation as either ‘Correct’ or ‘Incorrect’ for the two tasks above. Instructions were standardized, and edge cases were discussed during training rounds to ensure consistency. Results were compiled separately for each stage to evaluate performance progression across the pipeline.

For Job Title Identification, we evaluated the LLM’s performance using accuracy, precision, and recall.

1. Accuracy reflects the overall effectiveness in identifying job titles, indicating the proportion of both valid and invalid titles that were correctly identified.
2. Precision measures the correctness in identifying valid titles, i.e., the proportion of correctly identified valid titles out of all titles flagged as valid by the model.
3. Recall captures the model’s ability to find all valid job titles, i.e., the proportion of correctly identified valid titles out of all actual valid titles.

For Job Title Standardization, we assessed how accurately the LLM transformed valid titles into concise, professional forms. For example, converting ‘sumer intern’ to ‘Summer Intern’ is correct, while simplifying ‘accounting team’ to ‘Accountant’ is incorrect. The LLM was also expected to preserve hierarchical and specialization cues—for instance, changing ‘senior manager’ to ‘Manager’ or ‘IT manager’ to ‘Manager’ would be considered incorrect. Performance in this task was measured using accuracy, defined as the proportion of correctly standardized titles among all valid titles identified.

Across all four stages (JM1, JS1, JS3, and J3) the LLM consistently achieved strong performance. For “Job Title Identification”, the model attained an average accuracy of 98.5%, precision of 99.1%, and recall of 98.9%. For “Job Title Standardization”, it achieved an accuracy of 98.0%. These results demonstrate the model’s robustness in both identifying and standardizing job titles. Detailed results for each step are presented in Table 3.

**Table 3:** Validation Results Across Different Stages of the Cleaning Process.

		Accuracy	Precision	Recall
**JM1**	**Task 1**	0.984	0.987	0.996
	**Task 2**	0.977	—	—
**JS1**	**Task 1**	0.981	0.992	0.983
	**Task 2**	0.977	—	—
**JS3**	**Task 1**	0.984	0.992	0.990
	**Task 2**	0.985	—	—
**J3**	**Task 1**	0.989	0.991	0.988
	**Task 2**	0.982	—	—

This table presents accuracy, precision, and recall for Tasks 1 and 2 at various stages of data cleaning. Each row corresponds to a different processing stage (JM1, JS1, JS3, J3). Precision and recall are reported where applicable.

### Promotions Validation

To ensure the reliable validation of job transitions, we recruited annotators through Prolific [www.prolific.com] (Accessed in January 2025). The study received Institutional Review Board (IRB) approval under protocol HRPP-2024-137. The eligibility criteria required participants to be at least 30 years old and hold a Master’s degree or higher in one of the 24 sectors. This requirement aimed to ensure that the annotators had sufficient professional experience and contextual understanding of career progression within their sector.

**Fig. 6:**
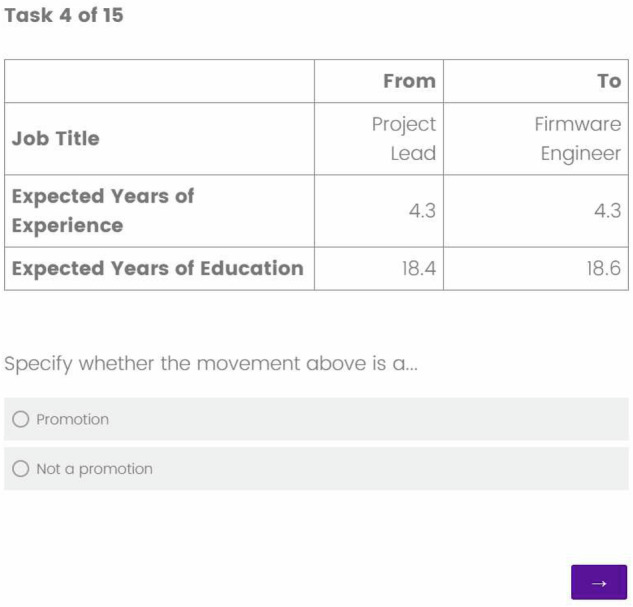
A Sample Survey Question for Job Transition Annotation. Participants assess whether the given transition, from one job title to another, is a promotion or not.

Each annotator was assigned job transitions based on their country of residence (United States or United Kingdom) and their industry sector, ensuring that they evaluated movements relevant to their expertise. Before beginning the annotation process, participants reviewed and accepted a consent form outlining the study’s objectives and confidentiality guidelines. To promote consistency in classification, all annotators were provided with detailed instructions prior to labeling. These instructions included standardized definitions of what constitutes a promotion, with an emphasis on upward movement in job responsibility, seniority, and required experience or education. During the annotation process, each annotator was presented with up to 15 job transitions and given the job titles, the average years of experience required for each position, and the average years of education required (For an example, see Fig. 6). Based on this information and the provided criteria, annotators were asked to determine whether each transition constituted a promotion.

To mitigate bias from the expectation that most transitions were promotions, we randomly reversed 50% of the transitions. That is, if a transition from A to B was likely to be a promotion, we showed annotators the reversed direction (B to A) half of the time. If the annotator marked the reversed transition as a demotion, we then confirmed the original A to B movement as a valid promotion. This approach helped balance the data and avoid annotation inflation. Additionally, each annotator was shown one obvious promotion relevant to their sector as an attention check. For example, annotators in the education sector were shown ‘Assistant Professor` to ‘Associate Professor`. Annotators who failed this check were excluded from the final analysis to ensure data quality and reliability.

A stratified sample of about 6,000 job transitions was validated, with 250 job movements randomly selected per sector (200 from the United States and 50 from the United Kingdom). In two sectors, Agriculture & Forestry and Consumer Services, the number of predicted promotions was smaller than the target validation sample size; in these cases, all predicted promotions were fully validated. Each job transition was independently evaluated by three different annotators, and a job transition was classified as a valid promotion or non-promotion if at least two out of three annotators agreed on its classification. The validation process resulted in an overall weighted accuracy of 86.2% for job transitions in the United States and 87.5% for those in the United Kingdom. These results indicate a high level of agreement among annotators, reinforcing the reliability of the validated promotion dataset. For sector-level accuracy results, see Table 4.

**Table 4:** Human validation accuracy for predicted promotions in the United States and the United Kingdom, reported per sector.

Sector	United Kingdom	United States
Accounting and Legal	98.0%	94.5%
Aerospace and Defense	80.0%	89.0%
Agriculture and Forestry	100.0%	94.9%
Arts Entertainment and Recreation	84.0%	87.5%
Biotech and Pharmaceuticals	86.0%	80.5%
Business Services	94.0%	86.0%
Construction Repair and Maintenance	82.0%	85.0%
Consumer Services	100.0%	85.1%
Education	84.0%	88.0%
Finance	92.0%	85.5%
Government	88.0%	81.0%
Health Care	92.0%	85.0%
Information Technology	86.0%	86.5%
Insurance	82.0%	83.0%
Manufacturing	86.0%	82.0%
Media	82.0%	85.0%
Nonprofit	88.0%	87.0%
Oil Gas Energy and Utilities	82.0%	87.5%
Real Estate	96.0%	83.5%
Restaurants Bars and Food Services	94.0%	96.4%
Retail	94.0%	84.5%
Telecommunications	92.0%	89.0%
Transportation and Logistics	84.0%	87.0%
Travel and Tourism	86.0%	88.0%
**Sector-Unweighted Accuracy**	**88.0%**	**86.4%**
**Sector-Weighted Accuracy**	**87.5%**	**86.2%**

Accuracy is based on stratified samples of 200 transitions per sector in the US and 50 per sector in the UK, with each transition evaluated by three expert annotators. The table also reports unweighted and weighted overall accuracy for each country; weighted overall accuracy accounts for the distribution of predicted promotions across sectors in the full dataset.

## Usage Notes

This dataset can be used both as a standalone resource and in conjunction with other data sources. Its flexible structure and high granularity make it well-suited for researchers studying labor markets, mobility patterns, and organizational hierarchies.

### Integration with External Datasets

While existing occupational datasets such as O*NET, the American Community Survey (ACS) PUMS, and wage records provide useful information about work contexts, job complexity, and demographic trends, they often lack details related to job title hierarchies. Our dataset fills this gap by offering large-scale, empirically validated promotion data and job titles as they are used in practice across millions of CVs.

Specifically, our dataset can be integrated with the following external resources:

- O*NET (Occupational Information Network): Our dataset includes crosswalks to O*NET Standard Occupational Classification (SOC) codes, enabling researchers to enrich job titles with ONET’s data on required skills, tasks, work context, and job complexity. However, while O*NET operates at the SOC level—aggregating many job titles into a single occupational category—our dataset retains title-level specificity, including hierarchical distinctions (e.g., “Analyst” vs. “Senior Analyst” vs. “Lead Analyst”), sectoral placement, and specialization level.
  Thus, O*NET and our dataset together offer a complementary view: O*NET characterizes the functional attributes of jobs and what they entail, while ours maps where they sit in the labor market and how individuals move between them.
- **ACS PUMS / IPUMS / Eurostat / Census data**: When merged with demographic surveys, our promotions dataset enables researchers to study how variables such as gender, race, age, education level, or region shape career progression. These integrations can shed light on inequalities in upward mobility and support the development of workforce policies.
- **Wage and compensation data (e.g., Glassdoor, Payscale, national labor statistics)**: By combining our dataset with wage records, researchers can assess whether promotions are associated with meaningful increases in pay. Analyses can be stratified by sector, geography, or demographic group to investigate whether compensation growth mirrors career advancement.
- **Job postings data (e.g., LinkedIn, Indeed, Glassdoor)**: The included job title mappings (raw  → cleaned  → generalized/simplified) provide a robust pipeline for cleaning and standardizing noisy titles from real-world job advertisements. This is especially useful for practitioners and data scientists working on tasks such as job classification, recommender systems, or labor market analysis. The extended mappings can also be used to train supervised machine learning approaches for job title normalization, especially in contexts where inconsistent or ambiguous titles hinder downstream analysis.

Examples of such integrations are demonstrated in the examples/ directory, which includes integration with (i) ACS PUMS and (ii) LinkedIn job postings.

### Standalone Applications

When used independently, the dataset enables in-depth analysis of career pathways and organizational hierarchies. One of its distinguishing features is the Specialization Index (*SI*), a metric designed to capture how embedded or generalized a job title is within specific sectors, and which provides a novel lens for understanding occupational structure. It is also composed of two interpretable components:

- Sector Exclusivity (*SE*), which measures widely a title is distributed across sectors. Titles with low SE are generalist roles (e.g., “Project Manager”), while those with high SE are tightly tied to a specific domain (e.g., “Actuarial Analyst”).
- Sector Dominance (*SD*), which reflects how prominent the title is within its primary sector, helping identify central vs. peripheral roles.

This index allows users to examine sector-specific vs. generalist roles, explore geographic variation in title specialization, study how certain occupations anchor industry-specific labor markets, and more. Unlike O*NET’s focus on task complexity or required education, the *SI* emphasizes how job titles circulate through sectors and reflects the real-world granularity of occupational usage.

The job title mappings also support title normalization efforts, especially in datasets plagued by inconsistent or idiosyncratic job titles. Researchers and practitioners can adopt these mappings directly or use them as training data to build their own classification models.

### Promotions Analysis and Network-based Research

The promotions component of the dataset is particularly valuable given the lack of empirical data on promotions in most public occupational datasets. Our data provides 32 thousand manually validated transitions across sectors in the U.S. and U.K., and 61 thousand statistically inferred promotions across six continents. We also provide metadata on each transition, including: Education Progression Score (*EPS*), Job Start Score (*JS*S), Predicted promotion probabilities.

This enables detailed analysis of career mobility, allowing researchers to identify typical promotion pathways, detect bottlenecks, and examine rare events such as “double jumps”—instances where individuals skip one or more intermediate job levels during advancement. Graph-based approaches, such as PageRank, can be applied to the directed promotion networks to infer job hierarchy structures or determine which titles exert the most centrality within promotion pathways. These methods allow for the creation of sector-specific organizational ladders or the identification of roles that act as gateways to leadership positions.

An additional layer is provided through interactive HTML-based promotion network visualizations in the network/ directories. These sector- and region-specific graphs allow users to visually explore common promotion pathways, identify high-centrality roles, and detect structural barriers to advancement. This network lens complements the individual-level transition data and supports structural analyses of labor mobility.

### Predictive Modeling

Beyond descriptive or exploratory studies, the dataset supports predictive modeling. Researchers can use the included promotion probabilities and job metadata to build classifiers that predict upward mobility, model career stagnation, or estimate the likelihood of job hopping. The logistic regression framework used to generate promotion scores can be adapted or extended, and the underlying features—such as education progression, sectoral specialization, and experience level—offer a strong basis for feature engineering.

In addition, the dataset was designed with user flexibility in mind:

- Promotion thresholds (e.g., the 0.9 probability cutoff used in our analysis) can be increased to define upward mobility more conservatively depending on the research context.
- Specialization Index (*SI*) thresholds (e.g., the mean value of 0.1) can be recalibrated overall or on a sector-specific basis to classify titles as specialized or generalist.
- Users can choose the level of job title granularity that best suits their analysis—opting for cleaned, generalized, or simplified versions of job titles depending on the task at hand.
- The components of *SI*: Sector Exclusivity (*SE*) and Sector Dominance (*SD*), can also be analyzed independently, offering researchers greater analytical flexibility. For example, if the research is confined to a single sector, *SD* alone is often sufficient, as it captures how central or peripheral a title is within that sector’s hierarchy. In contrast, for cross-sectoral analyses, *SE* becomes a valuable metric, as it quantifies how widely a job title is distributed across sectors, distinguishing generalist roles from those that are tightly bound to a specific domain. This modular design allows researchers to tailor the SI framework to the scale and scope of their inquiry.

## Data Availability

All Python code to rerun the promotion validation described in this report (Table 4 Fig. 3) can be found on figshare^46^ [10.6084/m9.figshare.28229633].
